# Contrasting Networks for Recognition Memory and Recency Memory Revealed by Immediate-Early Gene Imaging in the Rat

**DOI:** 10.1037/a0037055

**Published:** 2014-06-16

**Authors:** Cristian M. Olarte-Sánchez, Lisa Kinnavane, Eman Amin, John P. Aggleton

**Affiliations:** 1 School of Psychology, Cardiff University; 2 Neuroscience and Mental Health Research Institute, Cardiff University; 3 School of Psychology, Cardiff University; 4 Neuroscience and Mental Health Research Institute, Cardiff University

**Keywords:** recency memory, recognition memory, rat, immediate-early genes, network models

## Abstract

The expression of the immediate-early gene c-*fos* was used to compare networks of activity associated with recency memory (temporal order memory) and recognition memory. In Experiment 1, rats were first familiarized with sets of objects and then given pairs of different, familiar objects to explore. For the recency test group, each object in a pair was separated by 110 min in the time between their previous presentations. For the recency control test, each object in a pair was separated by less than a 1 min between their prior presentations. Temporal discrimination of the objects correlated with c-*fos* activity in the recency test group in several sites, including area Te2, the perirhinal cortex, lateral entorhinal cortex, as well as the dentate gyrus, hippocampal fields CA3 and CA1. For both the test and control conditions, network models were derived using structural equation modeling. The recency test model emphasized serial connections from the perirhinal cortex to lateral entorhinal cortex and then to the CA1 subfield. The recency control condition involved more parallel pathways, but again highlighted CA1 within the hippocampus. Both models contrasted with those derived from tests of object recognition (Experiment 2), because stimulus novelty was associated with pathways from the perirhinal cortex to lateral entorhinal cortex that then involved both the dentate gyrus (and CA3) and CA1 in parallel. The present findings implicate CA1 for the processing of familiar stimuli, including recency discriminations, while the dentate gyrus and CA3 pathways are recruited when the perirhinal cortex signals novel stimuli.

Recognition memory is the ability to discriminate novel from familiar stimuli. Recency memory is the discrimination of familiar stimuli by their relative distance in time; that is, temporal order memory. Despite obvious similarities, there is growing evidence that recognition memory and recency memory partly depend on different neural pathways. For example, lesion studies in rats consistently implicate the perirhinal cortex in both object recognition and object recency memory (Barker & Warburton, 2011a, 2011b; Brown & Aggleton, 2001; Ennaceur, Neave, & Aggleton, 1996; Mumby & Pinel, 1994; Win**z**ters, Saksida, & Bussey, 2008), yet the hippocampus and medial prefrontal cortex are only consistently implicated in object recency memory (Albasser et al., 2012; Barker, Bird, Alexander, & Warburton, 2007; Barker & Warburton, 2011a, 2011b; DeVito & Eichenbaum, 2011; Fortin, Agster, & Eichenbaum, 2002; Forwood, Winters, & Bussey, 2005; Hannesson, Howland, & Phillips, 2004,Hannesson, Vacca, Howland, & Phillips, 2004; Kesner, Gilbert, & Barua, 2002; Mumby, 2001). The present study sought, therefore, to compare the networks supporting these two forms of memory. To achieve this goal, the study used immediate-early gene (IEG) imaging as an indirect measure of neural activity (Chaudhuri, 1997; Guzowski et al., 2005; Herdegen, 1996; Tischmeyer & Grimm, 1999). The IEG c-*fos* was selected because the expression of this gene not only increases in perirhinal cortex when rats experience novel stimuli (Aggleton & Brown, 2005; Aggleton, Brown, & Albasser, 2012; Albasser et al., 2013; Albasser, Poirier, & Aggleton, 2010; Wan, Aggleton, & Brown, 1999, 2001; Warburton et al., 2005, 2003; Zhu, Brown, McCabe, & Aggleton, 1995, Zhu, McCabe, Aggleton, & Brown, 1996), but this expression is required for effective long-term recognition memory (Seoane, Tinsley, & Brown, 2012); that is, it has an integral role within this form of memory.

Behavioral tests of object recency for rodents typically involve a protocol in which there is a discrete intervening event that helps to separate the two items to be distinguished based on their temporal properties (Ennaceur, 2010; Mitchell & Laiacona, 1998). In such tests, the animal is introduced to object A then object B in the same apparatus but is removed from the apparatus between these two object presentations. Subsequently, the animal is placed back in the apparatus to select between objects A and B when they are presented together for the first time (e.g., Albasser et al., 2012; Barker et al., 2007; Hannesson, Howland, et al., 2004; Hannesson, Vacca, et al., 2004; Mitchell & Laicacona, 1998). This form of recency testing, in which the stimulus presentations include distinctive events that separate the items (see Templer & Hampton, 2013), can be compared with the ability to select between items previously presented in a single, continuous series; that is, without any specific intervening event (e.g., Agster, Fortin, & Eichenbaum, 2002; Fortin et al., 2002; Shaw & Aggleton, 1993). Reflecting the majority of published studies on object recency by rodents, the present study focused on recency discriminations when the objects are separated by time and by a distinct event (being removed from the apparatus).

Rather than give each rat a single recency memory test, which might not be sufficient to produce a measurable difference in c-*fos* expression, the rats received 20 recency tests prior to histological analysis (Experiment 1b). Consequently, for all tests in Experiment 1, each rat first explored 20 pairs of objects, where the objects in each pair were identical but differed from those in all of the other pairs (first sample phase). The second sample phase consisted of another 20 duplicate pairs of objects, which all differed from those in the first sample phase (Table 1). Each trial in the subsequent recency test consisted of pairs of nonidentical objects, one from the first sample phase, the other from the second sample phase. The bow-tie maze (Albasser, Chapman, Amin, Iordanova, Vann, & Aggleton, 2010; Albasser, Poirier, & Aggleton, 2010) was used for all behavioral testing because this apparatus makes it possible to deliver multiple trials without having to handle the rat.[Table-anchor tbl1]

A similar behavioral design was used for the recency control condition in Experiment 1b. The goal was to match the sensorimotor demands of the experimental group. For this reason, the first and second sample phases were identical to those used for the recency test (Table 1). The final test phase in the recency control condition involved the same objects as used for the recency test and, once again, each trial contained two different objects (Table 1). This time, however, the object pairings were selected to make their temporal properties indistinguishable. For this reason, each object pair in recency control consisted of two items from adjacent trials in the same sample phase. While objects to be discriminated in the recency test condition were separated by 110 min, those in recency control were separated by less than a minute. (In practice, this was just a few seconds.) In all other respects, the control protocol matched the recency memory condition (Table 1). Following either the recency or the control procedures, c-*fos* expression was quantified across multiple brain sites, with structural equation modeling (SEM) being used to test anatomically plausible patterns of functional connectivity.

Prior to recency testing, all rats (Experiment 1a; recognition control) were initially examined for their ability to recognize objects after the same retention delays as those proposed for the recency memory problem. Had the rats not been able to retain familiarity information in this recognition control test, then the subsequent recency task (Experiment 1b) could also effectively be seen as just a recognition test (novel vs. familiar). Finally, the network findings from Experiment 1b (Recency memory c-*fos*) were compared with additional c-*fos* derived networks in a new set of rats performing object recognition; that is, novel versus familiar discriminations (Experiment 2, Recognition memory c-*fos*). The data from Experiment 2 were combined those from a previous network study of recognition memory (Albasser, Poirier, & Aggleton, 2010) that used exactly the same methodology. Merging the data sets increased power and, thereby, made it possible to test more extended network models of recognition memory.

## Experiment 1: Recognition Control (1a) and Recency Memory c-***fos*** (1b)

### Materials and Methods

#### Animals

Subjects comprised 18 naïve, male, Lister Hooded rats (Harlan, Bicester, United Kingdom), which were housed in pairs under diurnal conditions (12-h light/dark). The rats were approximately 10 weeks old at the start of the study and weighed 277 to 355 g. During behavioral testing, the rats were food restricted but maintained at 85% of their free-feeding body weight. Water was available ad libitum. All experiments were performed in accordance with the United Kingdom Animals (Scientific Procedures) Act of 1986 and associated guidelines and were approved by local ethics committees (Cardiff University).

#### Apparatus

**Figure 1 fig1:**
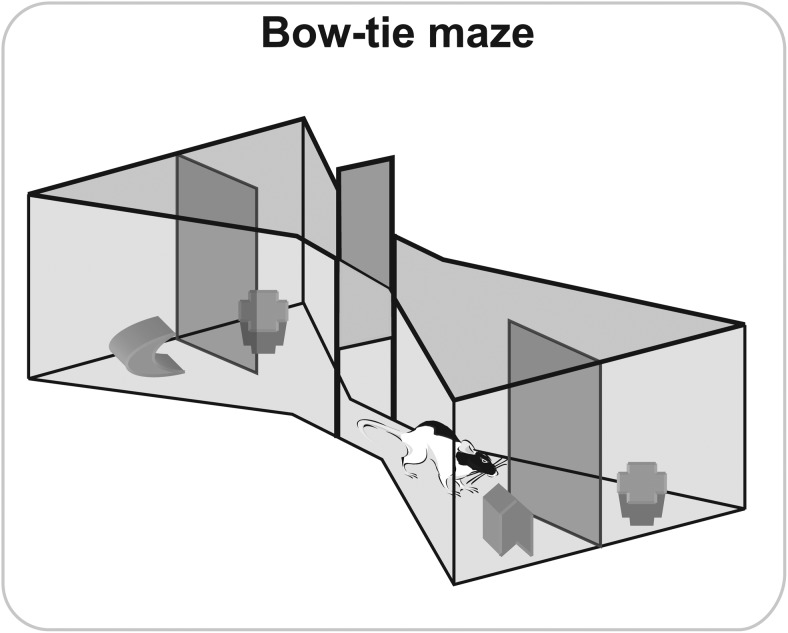
Schematic of the bow-tie maze with sliding door separating the two halves and objects covering the food wells. Figure adapted from Albasser et al. (2011).

The study used a bow-tie-shaped maze made with steel walls and a wooden floor (Figure 1). The maze was 1.2 m long, 0.5 m at its widest, and 0.5 m tall. Each end of the maze was triangular in shape, with the apices joined by a 0.12-m corridor. In the middle of the corridor was an opaque sliding door that divided the maze in half. Recessed into the floor, by the back wall of each triangular area, were two food wells, 3.5 cm in diameter, and 2 cm deep. These wells were separated by a steel wall that projected 15 cm into the maze from the center of the back wall.[Fig-anchor fig1]

#### Objects

Each experiment used separate collections of pairs of three-dimensional junk objects. Experiment 1a used 100 objects, whereas Experiment 1b used 80 objects (Table 1). Each pair was identical, but the pairs differed from one another in their color, shape, size, and texture. Any object with any obvious scent was excluded. All objects were large enough to cover a food well but light enough to be moved by a rat. The objects were cleaned with alcohol wipes after each session.

#### Behavioral testing

##### Pretraining

This phase lasted 7 days. By its completion, all rats would run from one side of the maze to the other and displace an object covering a food well to reach food rewards. On pretraining Day 1, pairs of rats were placed in the apparatus for 10 min, where they explored the maze freely and ate sucrose pellets scattered on the floor and in the food wells (45 mg; Noyes Purified Rodent Diet, Lancaster, NH). On Days 2 and 3, rats were placed individually in the maze for 10 min, where they were rewarded for shuttling between the goal areas at opposite ends of the maze. From Day 4, the central sliding door was used to control movement between the two sides of the maze. From Day 6, four pairs of different objects were introduced into the maze. An object was placed over a food well containing one sucrose pellet. Rats were rewarded for pushing these objects to access the food they uncovered. The pairs of objects used in pretraining were not used in later experiments.

##### General protocol

Both Experiments 1a and 1b involved two sample phases and one test phase (Table 1). The two protocols only differed in the final test phase. In both cases, each of the three phases contained 20 trials, each of 1-min duration. Each phase was separated by 90 min. At the beginning of the first sample phase, each rat was placed in one end of the maze, which was empty. The experimenter then lifted the central door so that the rat could run to the other side of the maze to begin Trial 1, where a pair of identical novel objects (A1 and A2) covered the two wells, each containing a single sucrose pellet. The rat was allowed to retrieve the pellets and freely explore the objects during the 1-min trial. The sliding door was then lifted so that the rat could run to the other side of the maze to begin Trial 2, where another duplicate pair of novel objects (B1 and B2) covered the two food wells. This sample phase protocol continued with pairs of identical novel objects, covering baited food wells, until 20 trials were completed. Rats were then placed in a dark, quiet holding room for 90 min, until the beginning of the next phase when they were returned to the bow-tie maze (Table 1).

The second sample phase was identical to that described previously, except that new duplicate pairs of objects were used on each of the 20 trials, such that 40 pairs of novel objects were seen by completion of the second sample phase. The rats were returned to the dark holding room for a further 90 min before the test phases for Experiments 1a and 1b began.

##### Experiment 1a (recognition control)

Following the two sample phases described previously, each trial in the test phase consisted of a pair of dissimilar objects over the two food wells. One object was familiar (from the first sample phase) while the other object was novel (Table 1). This test phase comprised 20 consecutive trials of 1 min each. As a consequence, recognition of the familiar object from the first sample phase involved a retention delay of 220 min. All objects (both novel and familiar) were baited with a single sucrose pellet, and the position of the novel object (left or right) was counterbalanced.

##### Experiment 1b (recency memory c-fos)

Pairs of rats from the same cage were randomly divided between two behavioral protocols (recency test and recency control). Testing began at least 5 days after completion of Experiment 1a.

##### Recency test

The two sample phases for the nine rats were exactly as described in the general protocol with new objects pairs. In the test phase, rats were now presented with two different, familiar objects; one object seen in the first (earlier) sample phase and a more recent object seen in the second sample phase (Table 1). The test phase began 90 min after completion of sample Phase 2. The pairs of objects were matched so that the object from Trial 1 of sample Phase 1 was paired with the object from Trial 1 of sample Phase 2, and so on. This meant that the objects from the two sample phases were separated by 110 min (Table 1). The test phase consisted of 20 trials, each 1 min. All objects were baited with a single sucrose pellet. Placement of the more recent object to the left or right side was counterbalanced. At test, items from the first sample phase were explored 220 min after their initial sample; that is, the same retention interval as used in Experiment 1a. Ninety minutes after completion of the final phase, the rats were perfused.

##### Recency control

The two sample phases for the nine rats were identical to those for the recency test group (Table 1). In the third phase, the rats were again presented with nonidentical pairs of objects that were seen previously in the sample phases. This time, the pairs of objects were taken from successive trials in the same phase. For example, in Trial 1 of the third phase, the objects presented were from Trial 1 and Trial 2 of the first sample phase, while in Trial 2 the objects presented were from Trial 1 and Trial 2 of the second sample phase (Table 1). The object pairings in the final test phase not only ensured that the order in which individual objects occurred was as closely matched as possible to that used in the recency test, but also ensured that the recency differences were particularly small because they were between objects that occurred in consecutive trials. Although each sample trial lasted 1 min, because the rats ran directly from the end of one sample trial to the next sample trial, the interval between successive objects was, in practice, often just a few seconds. The test phase again consisted of 20 trials of 1 min each. All objects were baited with a single sucrose pellet. Placement of the more recent object on the left or right was counterbalanced. Ninety minutes after completion of the test phase, the rats were perfused.

#### Analysis of behavior

All phases of both experiments were video-recorded. The time rats spent exploring the objects was measured by holding down a key on a computer linked to a timer. Object exploration was defined as directing the nose at a distance <1 cm from the object with the vibrissae moving and/or touching it with the nose or paws. Behavior that did not count as exploration included when rats sat on the object, if they used the object to rear upward with nose pointing at the ceiling, or if they chewed the object. From the timings of exploratory behavior, two measures of discrimination were calculated. Index D1 is the amount of time spent exploring the novel or older object, minus the time spent exploring the familiar or more recent object. The cumulative D1 is the sum of the D1 scores for all 20 trials. The second measure, D2 (Ennaceur & Delacour, 1988), compensates for individual differences in total exploration times. To calculate this index, the difference in time spent exploring the objects is divided by the total time spent in object exploration; that is, D1 is divided by total object exploration. Consequently, the D2 ratio can range between −1 and + 1. If the ratio is positive, the rat exhibits a preference for novel objects (recognition) or less recent objects (recency). The updated D2 score was recalculated after every trial using the cumulative amounts of exploration.

#### Immunohistochemistry

Following completion of the behavioral tests, rats were placed in a dark holding room for 90 min. This interval was selected because previous studies have shown that expression of Fos, the protein product of c-*fos*, peaks between 60 and 120 min after the inducing event (Bisler et al., 2002; Zangenehpour & Chowdhari, 2002). The rats then received a lethal overdose of sodium pentobarbital (60 mg/kg, intraperitoneal [IP], Euthatal, Rhone Merieux) and were transcardially perfused with 0.1M phosphate-buffered saline (PBS), followed by 4% paraformaldehyde in 0.1M PBS (PFA). The brains were removed and postfixed in PFA for 4 hr, then incubated in 25% sucrose at room temperature overnight on a stirrer plate.

The brains were cut in the coronal plane into 40-μm sections using a freezing microtome. A series of 1 in 4 sections was collected in PBS, then stained with cresyl violet. Other series were processed concurrently in pairs (one from each group). Sections were washed six times in 0.2% Triton-X 100 in 0.1M PBS (PBST), once in 0.3% H_2_O_2_ in PBST, then four times in PBST. Sections were then incubated in primary antibody solution, rabbit-anti-Fos diluted in PBST (1:3000; Ab-4, Calbiochem), for 48 hr at 4° C. Sections were washed four times in PBST, then incubated in secondary antibody solution, biotinylated goat-anti-rabbit (1:200; Vector Laboratories) diluted in 1.5% normal goat serum in PBST for 2 hr at room temperature. Sections were washed four times in PBST, then incubated in avidin-biotinylated horseradish peroxidase complex in PBST (Elite kit, Vector Laboratories) for 1 hr at room temperature. Sections were next washed four times in PBST, then twice in 0.05M Tris buffer. All previously mentioned washes were 10 min. Finally, diaminobenzidine (DAB Substrate kit, Vector Laboratories) was used to visualize the reaction, then stopped in cold PBS. Sections were mounted onto gelatin-coated slides, dehydrated, and coverslipped.

#### Fos-positive cell counts

Digital data from the regions of interest (ROIs) were captured in both hemispheres from four consecutive sections (each 120 μm apart) using a Leica DMRB microscope and an Olympus DP70 Camera. Immunopositive cells were counted using ANALYSIS^D software (Soft-Imaging Systems, Olympus, Southend, United Kingdom). This software selects and counts cells automatically, avoiding experimenter bias. In addition, the experimenter was blind to the group conditions. While stereological methods are essential to derive accurate absolute cell counts (Coggeshall & Lekan, 1996), the goal of the present study was to compare relative Fos-positive counts between areas and between the two conditions. For this purpose, automated cell counting is appropriate when certain conditions are met. These conditions include no systematic changes in the volume or packing of the neurons across the two groups along with random tissue sampling (Coggeshall & Lekan, 1996; Mura, Murphy, Feldon, & Jongen-Relo, 2004). These conditions should be met in the present study.

Counts of labeled nuclei in each ROI were determined by counting those nuclei (mean feret, a measure of particle size, of 4–20 μm) stained above a threshold of greyscale intensity that was above background levels. Counts with cortical regions were made in a frame area of 0.84 × 0.63 mm that enabled all laminae to be included in one image. Image montages of the hippocampus were used for the dentate gyrus, CA3 and CA1 fields. These montages were created from coronal sections at the septal, intermediate, and temporal levels of the hippocampus.

#### Regions of interest (ROIs)

**Figure 2 fig2:**
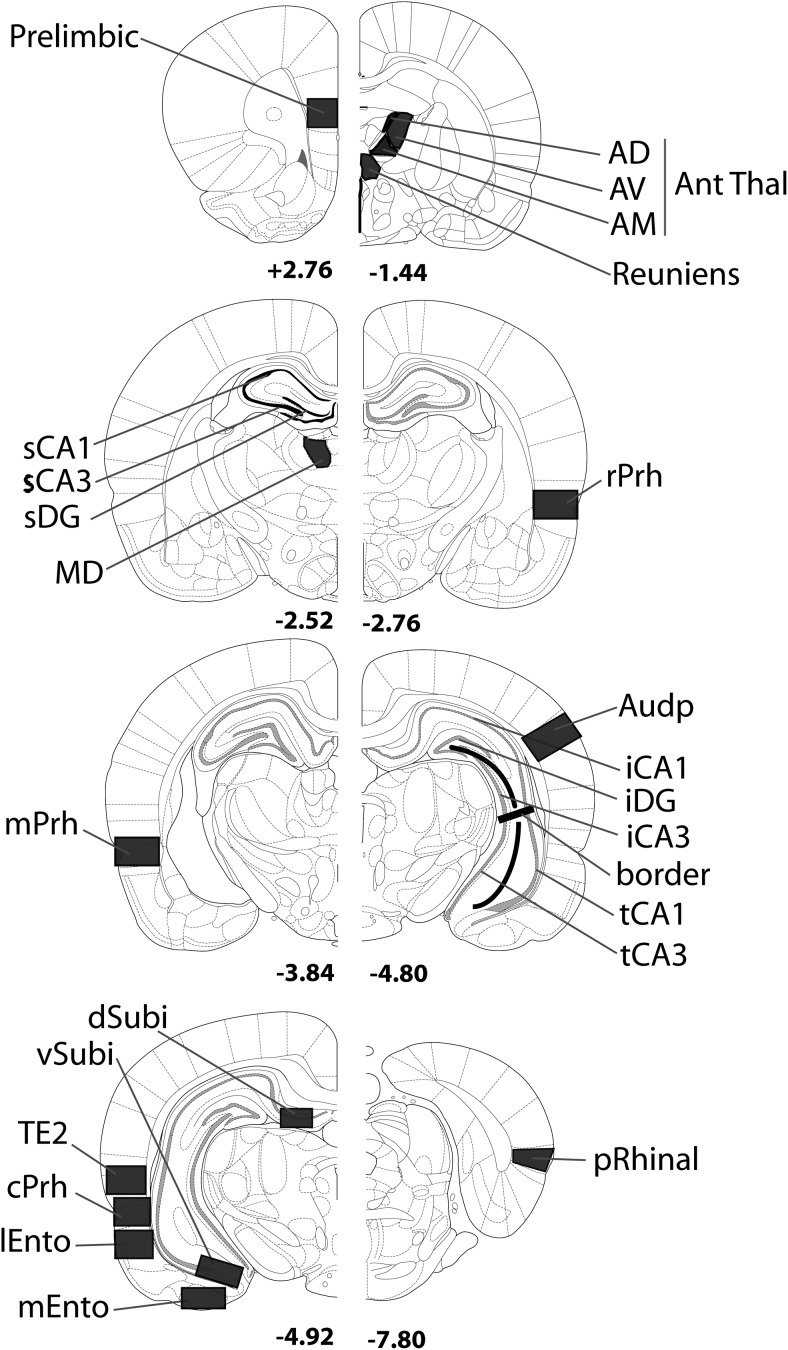
Regions of interest for c-*fos* analyses. AD = anterodorsal thalamic nucleus; AM = anteromedial thalamic nucleus; Ant Thal = anterior thalamic nuclei; Audp = primary auditory cortex; AV = anteroventral thalamic nucleus; CA fields = intermediate (i), septal (s) and temporal (t); DG = dentate gyrus; dSubi = dorsal subiculum; Hpc = hippocampus; lEnto = lateral entorhinal cortex; mEnto = medial entorhinal cortex; MD = medial dorsal thalamic nucleus; Prelimbic = prelimbic cortex; Prh = perirhinal cortex, caudal (c), mid (m) and rostral (r); pRhinal = postrhinal cortex; Reuniens = nucleus reuniens of thalamus; TE2 = area Te2; vSubi = ventral subiculum. The numbers refer to the distance (mm) from bregma. From *The Rat Brain in Stereotaxic Coordinates* (5th ed.) pp. 52, 85, 94, 96, 105, 113, 114 and 138 by Paxinos & Watson, 2005, New York, NY: Academic Press. Copyright, 2005 by Elsevier Academic Press. Adapted with permission.

The multiple ROIs are illustrated in Figure 2. Two brain atlases (Paxinos & Watson, 2005; Swanson, 1992) helped to verify the locations of brain areas, unless otherwise specified. The anterior–posterior (AP) coordinates (relative to bregma) in the descriptions that follow and in Figure 2 are from Paxinos and Watson (2005). The regions that follow reflect the groupings subsequently used in the statistical analyses of Fos counts.[Fig-anchor fig2]

##### Perirhinal cortex

The perirhinal cortex nomenclature and borders were taken from Burwell (2001). Separate counts were made in the rostral (from AP −2.76 to −3.84), mid (AP −3.84 to 4.80), and caudal (from AP −4.80 to −6.30) perirhinal cortex. The perirhinal cortex was also subdivided into areas 35 (ventral) and 36 (dorsal), making a total of six areas within the perirhinal cortex.

##### Entorhinal cortex and related areas

Separate cells counts were taken from the lateral and medial entorhinal cortices from sections near AP −4.92. Fos counts were also made in the postrhinal cortex, much of which corresponds to the caudal part of the area labeled as the ectorhinal cortex by Swanson (1992). In addition, cell counts were taken from the visual association area Te2, which is adjacent to area 36. This cortical area is interconnected with the postrhinal, perirhinal, and lateral entorhinal cortices and has previously been implicated in visual novelty detection (Albasser, Poirier, & Aggleton, 2010; Ho et al., 2011; Wan et al., 1999; Zhu et al., 1996).

##### Hippocampus

Hippocampal subfields (dentate gyrus, CA1 and CA3) were subdivided into their septal (dorsal), intermediate, and temporal (ventral) divisions (Bast, 2007). The septal hippocampus counts (dentate gyrus, CA3 and CA1) were obtained from sections near AP −2.52, whereas those for the intermediate hippocampus (dentate gyrus, CA1 and CA3) came from sections near AP −4.80. The border between the intermediate and temporal hippocampus corresponds to −5.0 dorsoventral from bregma (Paxinos & Watson, 2005). Within the temporal (ventral) hippocampus, counts were made in CA1 and CA3 fields at approximately AP −4.80. Additional cell counts were taken in both the dorsal and ventral subiculum (from around AP −4.92).

##### Frontal cortex and limbic thalamus

Fos-positive cell counts were made within the prelimbic region (from AP +3.20 to +2.76). Cell counts were also made in five thalamic nuclei that are directly interconnected with either the hippocampus or prefrontal cortex. These were the anterodorsal (AD), anteromedial (AM), anteroventral (AV), medial dorsal (MD) nuclei, and nucleus reuniens (Figure 2).

##### Auditory cortex

Counts of Fos-positive cells were made in the auditory cortex to provide an area where a null result might be expected if the behavioral tasks are well matched.

#### Statistical analysis

##### Behavior

For Experiment 1a, the final cumulative D1 and updated D2 scores were compared using two-sample *t* tests (two-tailed) for the sets of rats that would subsequently comprise the separate behavioral groups in Experiment 1b. Next, one-sample *t* tests (two-tailed), were applied to these final D1 and D2 scores to determine whether the indices of performance were significantly different from zero (chance level) for the two groups of rats. The same analyses were also applied to the behavioral data from Experiment 1b, with the addition that total cumulative levels of exploration were also compared between the two recency groups (two-tailed, two-sample *t* test). Additional paired sample *t* tests (two-tailed) were calculated on the behavioral measures for the recency control group to compare between trials involving objects from the first sample phase and those from the second sample phase (Table 1). For Experiment 1b, the behavior comparisons were within-subject to reflect the rat pairings in the subsequent IEG analysis.

##### IEG analyses

Initial comparisons used the raw counts of Fos-positive cells in the ROIs to make direct comparisons between the two recency groups. Individual rats from the two behavioral groups were paired, making these analyses within-subjects. Furthermore, subareas within the various ROIs (e.g., within the hippocampal formation and parahippocampal region) were first brought together in groups and then analyzed with an analysis of variance. This grouping procedure reduced the numbers of comparisons and so helps to protect against Type I errors. These groupings are described in the ROIs section.

The Fos counts for all individual areas were also correlated (Pearson product–moment coefficient) with all of the other areas, as well as with the behavioral indices of performance (D1, D2, total exploration), for each of the two groups. In view of the large number of individual areas counted (27 in total), some sites were again combined prior to these correlations to reduce the total numbers of comparisons. Examples include the three anterior thalamic nuclei, the subfield counts across different parts of the hippocampal AP axis (septal, temporal, intermediate), the dorsal and ventral subiculum, and areas 35 and 36 of perirhinal cortex.

#### Structural equation modeling (SEM)

SEM is a statistical methodology to evaluate interrelationships among variables (correlated or not) comprising an underlying theoretical structure. The term “structural equation model” refers to multiple-equation regression models representing supposed causal (and hence structural) relationships between variables, some of which may influence one another reciprocally. One feature is that it provides a method to test for possible directions of effect. This same approach also makes it possible to evaluate the viability of network (model) dynamics (Friston, Frith, & Frackowiak, 1993; Jenkins, Amin, Harold, Pearce, & Aggleton, 2003; McIntosh & Gonzalez-Lima, 1991; Poirier, Amin, & Aggleton, 2008). To use this approach, it is assumed that all models should be based on established patterns of connectivity between the ROIs. To contrast activation patterns across groups (recency test, recency control), the same network can be used to compare levels of fit. (The term fit refers to the ability of a model to reproduce the data, i.e., the variance–covariance matrix.) In addition, optimal network models can be created for each group from the covariance matrices representing the associations among the Fos counts in the different ROIs. The specialized SEM software Amos 18 (IBM) was used.

Because different fit indices can be more or less sensitive to various parameters derived from the model (Fan, Wang, Thompson, & Wang, 1999; Hu & Bentler, 1998), it is advisable to report several indices. The indices aim to test statistically the explanatory power of the models (similar to the *F* test for an analysis of variance [ANOVA]). Several measures of goodness-of-fit are reported in the present study. The first one is a nonsignificant χ^2^, which gives the only binary fit/no-fit decision for a model. Two more measures give an index of the degree of fit of a model to the data. The first measure is the Comparative Fit Index (CFI), which indicates the proportion of variance accounted for, based on the comparison of the proposed model to an independent model, in which no anatomical regions are connected. This independent model has the poorest fit, and a high index value means that the tested model is opposite to the independent model, which indicates a good fit. The second measure used is the root mean square error of approximation (RMSEA), which provides an index of absolute fit. This index is the mean lack of fit per degree of freedom.

The CFI and RMSEA are appropriate indexes for this study because they are both recommended for their good performance with small sample sizes (Fan et al., 1999; Hu & Bentler, 1998), thus each helps to overcome the power limitations of the current study. In summary, along with a nonsignificant χ^2^ and a ratio of the χ^2^ to the degrees of freedom of <2, a good-fitting model of the data was considered to have a CFI ≥ 0.95, and RMSEA ≤ 0.05 (good) or ≤ 0.08 (acceptable; Fan et al., 1999; Tabachnik & Fidell, 1996).

Groups were compared by stacking their Fos data on the same model to test for group differences. For this comparison, the structural weights of all paths in the model are constrained to be equal across groups and the fit is compared with that of the model in which the structural weights of all paths are free to vary between the groups. If the model fit when the paths are unconstrained is significantly better, as determined by a χ^2^ difference test, this indicates that the data vary among the groups (Protzner & McIntosh, 2006).

### Results

#### Experiment 1a (recognition control)

**Figure 3 fig3:**
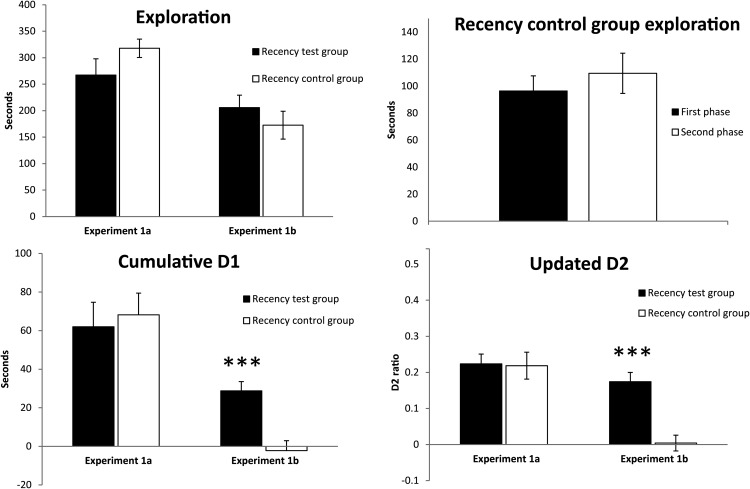
Experiment 1a and 1b: Behavioral measures of object recognition and recency memory. The top left graph shows the mean total time spent exploring objects in the test phase of Experiment 1a and 1b. The top right graph illustrates the exploration of the recency control group in Experiment 1b divided into exploration of objects first seen in sample Phase 1 and objects first seen in sample Phase 2. The bottom left graph shows the total difference in time spent exploring novel objects over familiar objects (Experiment 1a) or recent objects over less recent objects (Experiment 1b) across the 20 trials (cumulative D1). The bottom right graph represents the same data as the bottom left graph, but now the discrimination scores are expressed as the Updated D2 ratios (see Methods). All graphs show the mean performance ± *SEM*. Note that for Experiment 1a, the group names refer to how the rats were subsequently allocated for Experiment 1b. *** *p* < .001.

Comparisons between the two sets of rats that would subsequently form the recency test group and the recency control group showed that there were no significant differences for either the cumulative D1, *t*(16) = 0.37, *p* = .72, or final, updated D2, t(16) = 0.11, *p* = .92. It is important that both groups of rats displayed a clear preference for novel over familiar objects in the test phase (Figure 3). Of particular note is that the future recency test group was above chance for both discrimination measures, one-sample *t* test; D1 *t*(8) = 4.87, *p* = .001; D2 *t* test; *t*(8) = 8.25, *p* < .001. Likewise, the same discrimination measures for the future recency control group were above chance, D1 *t*(8) = 6.06, *p* < .001; D2 *t*(8) = 5.84, *p* < .001.[Fig-anchor fig3]

#### Experiment 1b (recency memory ***c-fos***)

##### Behavior

As expected, the recency test group had superior recency discrimination scores to those of the recency controls, as measured by both the cumulative D1, *t* test; *t*(8) = 4.48, *p* = .002, and updated D2, *t* test; *t*(8) = 3.80, *p* = .005, scores (Figure 3). The recency test group successfully discriminated objects in the first sample phase from those in the more recent, second sample phase, one-sample *t* test; D1 *t*(8) = 5.67, *p* < .001; D2 *t*(8) = 6.42, *p* < .001. In contrast, the recency control group failed to discriminate between objects that were temporally adjacent in the same series, one-sample *t* test; D1 *t*(8) = 0.40, *p* = .70; D2 *t*(8) = 0.17, *p* = .87. Finally, there was no group difference in total exploration times, *t*(8) = 0.82, *p* = .43 (Figure 3).

In view of their status, it is important to test whether the recency control group showed any differential behavior reflecting the temporal properties of the stimuli. Using data from the test phase only, it was possible to separate the final series of trials into those involving objects from the first sample phase (odd numbered trials) and those from the second sample phase (even numbered trials). No difference was found in total exploration, paired-sample *t* test; *t*(8) = 1.51, *p* = .17. Likewise, in neither subset of trials could the control rats discriminate the objects based on their relative recency, nor did the recognition score (D2) differ for these two subsets of trials, paired-sample *t* test; *t*(8) = 0.31, *p* = .77.

##### c-fos activation: Group differences and correlation data

**Figure 4 fig4:**
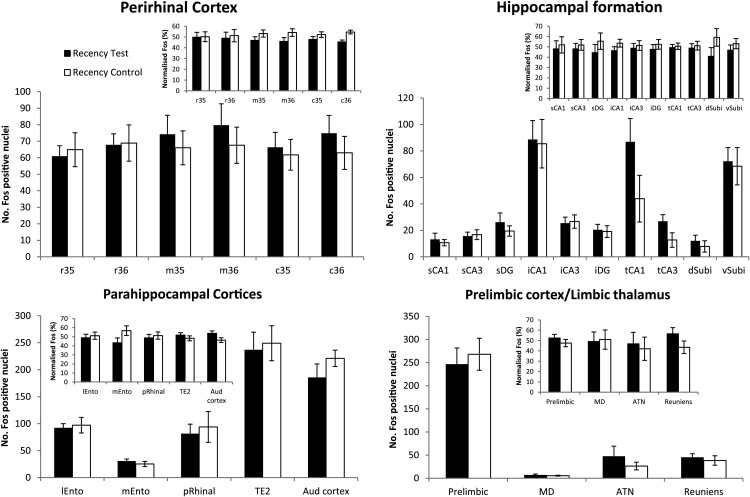
Counts of Fos-positive cells in regions of interest following the two behavioral conditions in Experiment 1b. Filled bars represent the recency test group and unfilled bars represent the recency control group. Inset histograms represent normalized (mean number of activated neurons in a given animal for a given site divided by the combined mean of the two animals in each matched pair expressed as a percentage) fos counts for each region. ATN = anterior thalamic nuclei; Audp = primary auditory cortex; CA fields = intermediate (i), septal (s) and temporal (t); DG = dentate gyrus, intermediate (i) and septal (s); dSubi = dorsal subiculum; lEnto = lateral entorhinal cortex; mEnto = medial entorhinal cortex; MD = medial dorsal thalamic nucleus; Prelimbic = prelimbic cortex; pRhinal = postrhinal cortex; reunions = nucleus reuniens; TE2 = area Te2; vSubi = ventral subiculum, areas 35 and 36 caudal (c), mid (m) and rostral (r). Data are presented as group mean ± *SEM*.

The counts of Fos-positive cells in the two behavioral conditions rarely differed in the various ROIs (Figure 4). Attention, therefore, focused on two sets of correlations. The first set of correlations concerned an area’s Fos count and the behavioral performance (D1, D2, total exploration) of each group (Table 2). To limit the numbers of correlations and to help reduce variance, the subfield data for the septal, intermediate, and temporal subregions of the hippocampus were combined. Likewise, the separate counts from areas 35 and 36 were combined to give counts for the rostral, mid, and caudal parts of the perirhinal cortex (Table 2). The full set of correlations is provided online in Supplemental Table 1.[Fig-anchor fig4][Table-anchor tbl2]

One concern is that the remaining multiple correlations will still increase the risk of Type I errors. For this reason, it is notable in the recency test group that of the 16 sites examined (Table 2), the Fos counts correlated (*p* < .05) with D2 scores in nine sites and with D1 in eight sites. Far fewer sites in the recency control group had Fos counts that correlated with either D2 or D1 (maximum of two), though these correlations in the control group are more difficult to interpret given that the recency memory scores in this group were close to zero. It is, therefore, particularly interesting that the opposite group pattern was seen for total exploration levels. None of the 16 sites had Fos counts that correlated with total exploration in the recency test group, but there was a significant correlation in five sites for the recency control group.

The second set of correlations concerns the interarea Fos scores within each of the two groups (Table 2; Supplemental Table 1 provides the correlations for all of the individual subareas counted, i.e., before some of the hippocampal and perirhinal subareas were grouped). These tables show probability levels uncorrected for multiple comparisons because the individual correlations are of limited significance. Rather, the anatomical constraints on the SEM analysis and their overall fit indices help to compensate for the Type I errors inherent in the multiple correlations that comprise the model (Tables 1 and 2). In view of this same issue, it is important that any model must match established patterns of connectivity between the ROIs; that is, potential models are constrained.

##### Perirhinal cortex

**Figure 5 fig5:**
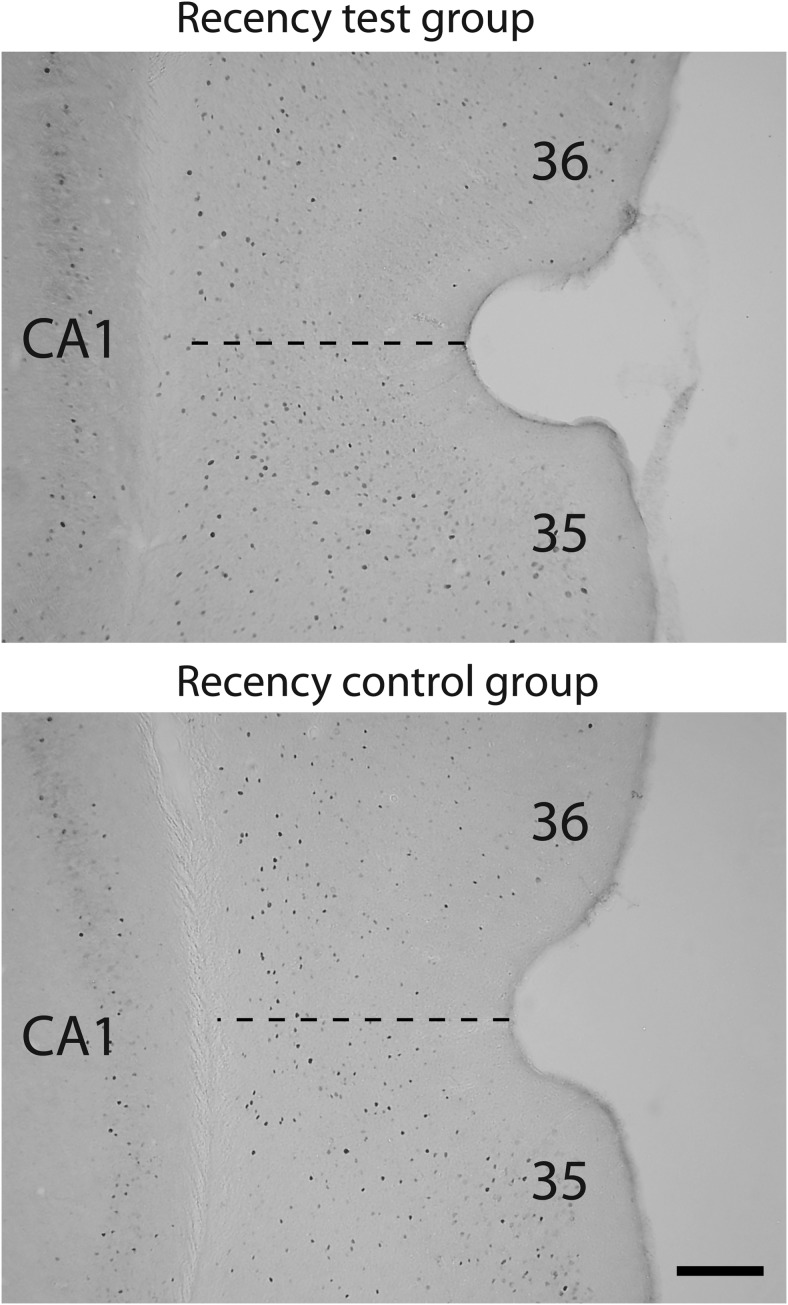
Photomicrograph showing Fos-positive cells in the perirhinal cortex (coronal section) from rats in the recency test (top panel) and recency control (bottom panel) groups. The areas shown are caudal perirhinal cortex (area 35 and 36) and hippocampal field CA1. Scale bar 200 μm.

Comparisons involving the six subareas (see ROI section) found no significant differences in the numbers of Fos-positive cells in the recency test and control groups (*F* < 1, interactions *F* < 1; Figures 4 and 5). For the recency test group, there were significant correlations between the Fos counts and the D1 index in rostral areas 35 and 36 (area 35 *r* = .76, *p* = .017; area 36 *r* = .67, *p* = .048), but no significant correlations were found with D2 (*p* > .1). Similarly, mid area 35 and 36 correlated with D1 (area 35 *r* = .80, *p* = .01; area 36 *r* = .69, *p* = .039), but again no significant correlations with D2 (area 35 *p* = .205; area 36 *p* = .056). However, a more consistent effect was seen in caudal 35 and 36 where c-*fos* counts significantly correlated with D1 (area 35 *r* = .80, *p* = .01; area 36 *r* = .72, *p* = .027) and with D2 (area 35 *r* = .79, *p* = .012; area 36 *r* = .88, *p* = .002). No comparable D1 or D2 correlations were found for the recency control group. The only significant correlation in this group was between caudal area 35 and total exploration (*r* = .72, *p* = .03).[Fig-anchor fig5]

##### Entorhinal cortex and related cortical areas

Among these areas, there was no evidence that the total counts of Fos-positive neurons differed between the experimental and control groups (*F* < 1), nor was there a ROI by group interaction (*F* < 1; Figure 4). There were, however, significant correlations between discrimination performance by the recency test rats and their Fos counts in the lateral entorhinal cortex (D1 *r* = .72, *p* = .029; D2 *r* = .76, *p* = .018) and in the medial entorhinal cortex (D1 *r* = .77, *p* = .014; D2 *r* = .72, *p* = .03). In the postrhinal area, a significant correlation was also found with D2 (*r* = .68, *p* = .045). Finally, the recency test group also had a significant correlation between Fos protein counts in area Te2 and the updated D2 ratio (*r* = .77, *p* = .014). The recency control group presented a very different picture because none of the parahippocampal cortical areas correlated with the discrimination parameters D1 or D2 (presumably reflecting the very low D1 and D2 scores). This group did, however, show a significant correlation between the Fos counts in the postrhinal area and total exploration (*r* = .69, *p* = .04).

##### Hippocampus (septal, intermediate, and temporal)

No significant differences were found in the total number of Fos-positive cells between the two behavioral groups in the septal, intermediate, and temporal CA1, CA3, or DG fields (*F* < 1), nor was there an interaction between region and group (*F* < 1; Figure 4). For the recency test group, significant positive correlations were found between D2 and CA1 Fos counts (*r* = .79, *p* = .011) but not D1 (*r* = .57, *p* = .106). Both discrimination indices correlated with Fos counts in CA3 (D1 *r* = .67, *p* = .049; D2 *r* = .68, *p* = .045). In the same group, neither D1 nor D2 correlated significantly with the dentate gyrus counts (D1 *r* = .39, *p* = .29; D2 *r* = .60, *p* = .09). The recency control group failed to show any significant correlation between discrimination behavior and Fos counts. (Note that the correlation data here refer to the subfield counts summed across the septal, intermediate, and temporal hippocampus.)

##### Subiculum

There was no evidence of a Fos count difference between the two behavioral groups (*F* < 1) in the subiculum (ventral subiculum, dorsal subiculum), nor was there an interaction between the behavioral groups and the ROIs (*F* < 1; Figure 4). However, for the recency test group, the ventral subiculum Fos counts showed significant correlations with both D1 (*r* = .78, *p* = .013) and D2 (*r* = .88, *p* = .002). There was also a borderline significant correlation between D1 and the dorsal subiculum Fos counts (*r* = .66, *p* = .052), but not for the D2 index (*r* = .55, *p* = .122). When the ventral and dorsal subiculum Fos counts were combined the correlations with D1 and D2 remained significant (*p* ≤ .009 for both). In contrast, analyses using the recency control data found no significant correlations for either the ventral or dorsal subiculum with D1 or D2, nor were these correlations significant when the counts for the two areas were combined (all *p* > .05).

##### Prelimbic cortex and limbic thalamus

The Fos scores for the three anterior thalamic nuclei were first combined as their individual counts were typically very low. The ROIs were the anterior thalamic nuclei (ATN), the MD thalamic nucleus, nucleus reunions, and the prelimbic cortex (Figure 4). The counts of Fos-positive cells did not differ between the two behavioral groups (*F* < 1), and there was no interaction (*F* < 1). The Fos count in nucleus reuniens correlated significantly with D1 (*r* = .72, *p* = .030) in the recency test group; no other significant correlations were found between the behavioral measures (D1 and D2) and the Fos counts in the other ROIs in the recency test group. There was a significant negative correlation in the recency control group between MD counts and both D1 (*r* = −0.76, *p* = .019) and the updated D2 (*r* = −0.70, *p* = .035) discrimination indices. In addition, the MD counts in this group significantly correlated with exploration (*r* = .91, *p* = .001). Also, in the recency control group the ATN Fos counts correlated significantly with D1 (*r* = −0.72, *p* = .029) and total exploration (*r* = .91, *p* = .001), but not with updated D2 (*r* = .59, *p* = .092). Finally, the Fos counts in nucleus reuniens and the prelimbic cortex only correlated significantly with total exploration (*r* = .70, *p* = .045; *r* = .69, *p* = .039, respectively) in the recency control group (D1 and D2, *p* > .4).

##### Auditory cortex

No differences were found between the two groups in this area, *F*(1,8) = 2.26, *p* = .171 (Figure 4). There was, however, a positive correlation between c-*fos* activation and the D2 ratio in the recency test group (*r* = .88, *p* = .002). No other significant correlations were found in the recency control group.

#### SEM

The Fos counts from areas 35 and 36 (perirhinal cortex) were combined, but the anterior, mid, and posterior perirhinal regions were kept separate because previous studies have identified the particular significance of caudal perirhinal cortex for visual recognition (Albasser, Davies, Futter, & Aggleton, 2009, Albasser, Poirier, & Aggleton, 2010). Within the hippocampus, the septal, intermediate, and temporal parts of CA1, dentate gyrus, and CA3 were combined prior to testing for network models because preliminary analyses based on the separate results from each division (septal, intermediate, or temporal) failed to create acceptable models. The counts from the three anterior thalamic nuclei were summed because the individual scores were low. Finally, the dorsal and ventral subiculum were combined but failed to yield models with good fit. Consequently, only the dorsal subiculum was used to create the models. It should be noted that it is the dorsal subiculum that principally projects to the anterior thalamic nuclei (Wright et al., 2013), and this same subicular subdivision was selected in previous hippocampal network studies of object recognition (Albasser, Poirier, & Aggleton, 2010).

##### Recency test group

**Figure 6 fig6:**
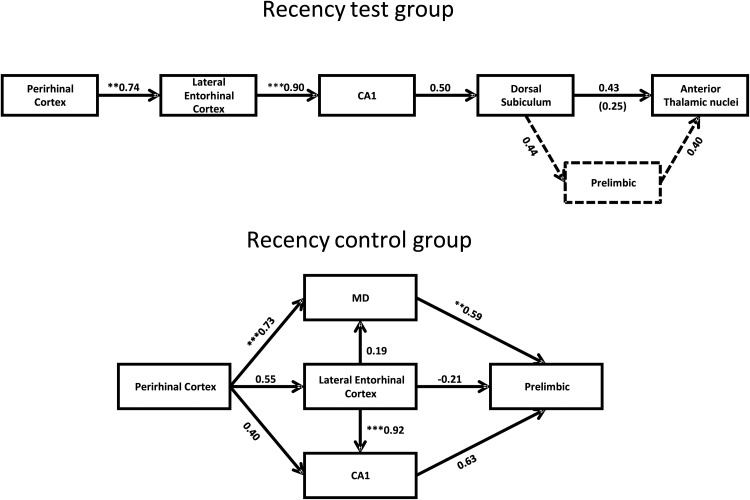
Schematic depiction of the networks with best fit for the recency test (upper) and recency control (lower) groups (Experiment 1b). Note that the dashed pathways involving the prelimbic cortex have been added to the recency test model because these provide a further model with good fit. The number in brackets is the path coefficient when the prelimbic cortex is added to the recency test model. MD = medial dorsal thalamic nucleus. ** *p* < .01. *** *p* < .001.

From the interarea correlations, it was possible to generate two very closely related models with good fit (Figure 6, upper), the only difference being whether prelimbic cortex was added to the network. The first network was a simplex (serial) model involving caudal perirhinal cortex and successive projections to lateral entorhinal cortex, CA1, dorsal subiculum, and the anterior thalamic nuclei (χ^2^ = 4.57, *df* = 6, *p* = .60; CFI = 1, RMSEA = 0.00). The second model (Figure 6, upper) involved additional projections from the dorsal subiculum to the prelimbic cortex and from the prelimbic cortex to the anterior thalamic nuclei (χ^2^ = 7.83, *df* = 9, *p* = .55; CFI = 1, RMSEA = 0.00). In both models, there were significant pathways from caudal perirhinal cortex to lateral entorhinal cortex (*p* = .002) and from lateral entorhinal to CA1 (*p* < .001). Also, as noted previously, there were significant correlations between D1 and D2, with Fos counts in the perirhinal cortex, lateral entorhinal cortex, CA1, and CA3.[Fig-anchor fig6]

A third acceptable model (not shown) again involved projections from perirhinal cortex to lateral entorhinal cortex, thence to CA1, with CA1 projecting to both prelimbic cortex and the dorsal subiculum, and both of these sites projecting to the anterior thalamic nuclei (χ^2^ = 9.23, *df* = 9, *p* = .42; CFI = 0.99, RMSEA = 0.058). None of the acceptable models for the recency test group involved the entorhinal projections to either dentate gyrus or CA3.

##### Recency control group

Only one acceptable model involving parahippocampal and hippocampal regions could be derived (Figure 6, lower). Like the recency test group, the model for the recency control group again included the caudal perirhinal area, the lateral entorhinal area, and CA1, but in addition the model incorporated the prelimbic cortex and the MD thalamic nucleus. The resulting network created a model with good fit (χ^2^ = 1.39, *df* = 3, *p* = .71; CFI = 1, RMSEA = 0.00). Three of the pathways involved in the models were significant; caudal perirhinal to MD (*p* < .001), lateral entorhinal to CA1 (*p* < .001), and the pathway from MD to prelimbic area (*p* = .003).

#### Comparison between the models for the recency test and control groups

A stacking procedure was undertaken between the recency test and recency control groups. Initially, the data from these groups were stacked on the simplex model found to be optimal for the recency test group; the structural weights of each of the paths were constrained such that they had to have the same value in both groups; that is, setting the corresponding pathways in each of the groups to be identical. There was no significant difference between the model in which the structural weight of the paths were constrained to be the same and the model in which they were free to vary, χ^2^(4) = 4.13, *p* = .39. This indicates that the data from both groups fit this model. This is not necessarily surprising considering both groups are exploring objects that are familiar because of a single previous exposure and the pathways from perirhinal cortex to lateral entorhinal cortex and then to CA1 are a component of both optimal models. However, the groups do differ from one another because the optimal models for each group are different (Figure 6). Furthermore, when the same stacking procedure is carried using the recency control optimal model, a significant difference is found between the model in which the structural weights are free to vary and the model in which they are constrained to be the same, χ^2^(8) = 19.6, *p* = .012. This illustrates that the Fos data from the recency test group does not fit the recency control group model.

## Experiment 2: Recognition Memory ***c-fos***

Experiment 1 sought to determine the patterns of c-*fos* activation associated with recency discriminations. Experiment 2 examined the patterns of c-*fos* activity when rats chose between novel and familiar objects (recognition memory). The design closely follows that of Albasser, Poirier, and Aggleton (2010).

### Materials and Methods

#### Animals

Subjects were eight adult (weight between 309 and 438 g) male Lister Hooded rats (Charles River, United Kingdom), housed in pairs under diurnal conditions (12-h light/dark). Two months before the present study, the rats completed an object recognition experiment in the bow-tie maze in which the numbers of food pellets placed under an object and the levels of food restriction conditions were manipulated. During the present study, the rats were again food restricted, being maintained up to 85% of their free-feeding body weight. Water was available ad libitum. The experiment was performed in accordance with the United Kingdom Animals (Scientific Procedures) Act of 1986 and associated guidelines and was approved by local ethical committees.

#### Apparatus

Testing was again in the bow-tie maze (Figure 1). The study used 142 identical pairs of three-dimensional objects, differing in shape, texture, and size. Many of these objects were the same objects used in Experiments 1a and 1b. The objects were cleaned with alcohol wipes after each session.

#### Behavioral testing

##### Pretraining

The procedure matched that used for Experiment 1, although pretraining occurred in a different testing room to that used for the later test sessions.

##### Testing protocol

All rats received 12 training sessions followed by one test session over 7 consecutive days (two sessions per day for the first 6 days, one test session on the final day). Each session comprised 20 trials. To start a session (Trial 1; Table 1), the rat was placed in one arm of the bow-tie maze. This arm contained a single object (A_1_) that covered a food reward. After 1 min, the central door was raised and the rat could now run to the other end of the maze, which contained two objects (A_2_ and B_1_). One object was new (B_1_), the other familiar from the previous trial (A_2_). Both objects covered a single sucrose pellet, which the rat retrieved. After one minute, trial two finished and the central door raised. The animal was now free to run to the other end of the maze (trial three), where it was again confronted with two objects, C_1_ (novel) and B_2_ (familiar from the previous trial). This running recognition procedure continued for 20 trials (see Table 1 for schematic). The animals were placed in a dark room for 30 min before each session, and after every session the animals were placed back in the same dark room for a further 30 min.

A different set of 20 objects was used for each of the first six sessions. This set of 120 objects was then reused for the remaining six sessions (Sessions 7 to 12); however, the order and pairing of objects were changed. Novel objects were placed to the left or right according to a counterbalanced sequence. On the final test day (Session 13), the rats were trained as described previously but received a totally new set of 20 pairs of objects. Following completion of the object recognition test, each rat was placed in the dark holding room for 90 min and then perfused.

#### Immunohistochemistry and cell counting

The methods were the same as those for Experiment 1, including the data analyses.

#### c-***Fos*** activation: ROIs

The only perirhinal regions analyzed in this experiment were caudal areas 35 and 36 because this combined area was used for the network models in Experiment 1. Furthermore, this region of the perirhinal cortex was used by Albasser, Poirier, and Aggleton (2010) to create SEMs associated with object recognition. Fos counts were also made in the lateral entorhinal cortex, medial dorsal and anterior thalamic nuclei, and the primary auditory cortex. As in Experiment 1b, separate counts were made in the septal, intermediate, and temporal parts of the various hippocampal subfields. The correlations based on the summed counts for these areas are shown in Table 3.[Table-anchor tbl3]

### Results

#### Behavior

The eight rats showed a strong preference for the novel objects. Their mean D1 (74.17 ± 7.23 SEM) and D2 (0.25 ± 0.02) scores were significantly higher than chance, one-sample *t* test; D1, *t*(7) = 10.26, *p* < .001; D2, *t*(7) = 14.36, *p* < .001.

#### c-Fos activation: Correlations with behavior

For this cohort of eight rats, none of the Fos counts in the ROIs showed a significant correlation with D1, D2, or total exploration.

#### c-***Fos*** activation: Interarea correlations

Table 3 (upper right) shows the correlations between the Fos counts for the various ROIs. It can be seen that there are clusters of correlations within the parahippocampal region (plus area Te2) and within the various hippocampal subfields. The same table (lower left) shows the corresponding correlations when data from Albasser, Poirier, and Aggleton (2010) are added. (Supplemental Table 2 provides the correlations for all of the individual subareas counted within the hippocampus and perirhinal cortex, i.e., before these subareas were summed).

#### SEM

As in Experiment 1, preliminary analyses used combined hippocampal divisions (septal, intermediate, temporal) to create models. The combined septal, intermediate, and temporal hippocampal counts did not, however, create plausible models because the fit indices were poor. Therefore, individual hippocampal divisions were examined. These analyses focused on the septal hippocampus because the same division formed good models in a previous study of recognition memory that used the same behavioral and imaging methods (Albasser, Poirier, & Aggleton, 2010). Hence, we were able to test the same models with the eight rats in the present study and also combine that data with the findings from the previous study (Albasser, Poirier, & Aggleton, 2010).

**Figure 7 fig7:**
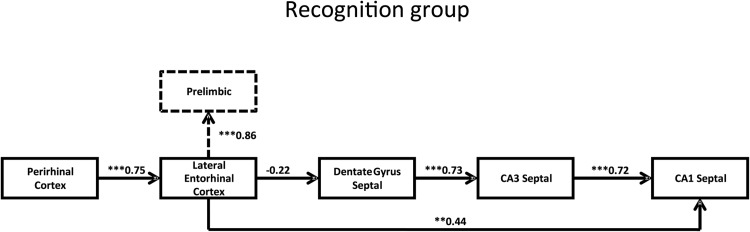
Structural equation model for recognition memory. The figure shows the network with best fit using combined data from Experiment 2 and from Group Novel in Albasser, Poirier, & Aggleton (2010), because the rats were trained and analyzed using the same methodology. The dashed pathways illustrate an additional acceptable model in which the prelimbic cortex has been added. ** *p* < .01. *** *p* < .001.

The best model using the combined data from the present recognition group and the corresponding data from Albasser, Poirier, and Aggleton (2010) is shown in Figure 7. This model is almost identical to that derived by Albasser, Poirier, and Aggleton (2010), except that in this case, area Te2 has been removed. Despite the finding that Fos counts in Te2 and caudal perirhinal cortex were highly correlated (*p* = .005, Table 3), the addition of Te2 did not yield a superior model. The object recognition activity network for the combined data involved the projections from caudal perirhinal cortex to the lateral entorhinal cortex, to septal DG, then to septal CA3 and on to septal CA1, with an additional direct pathway from lateral entorhinal cortex to septal CA1 (Figure 7). The model, which had good fit indices (χ^2^ = 4.87, *df* = 5, *p* = .43; CFI = 1.00; RMSEA = 0.00), included four significant pathways (all *p* ≤ .004). These pathways are from perirhinal cortex to lateral entorhinal cortex, dentate gyrus to CA3, CA3 to CA1, and lateral entorhinal to CA1. There is also an alternative model with acceptable fit indices (χ^2^ = 8.67, *df* = 9, *p* = .47; CFI = 1.00; RMSEA = 0.00) that could be created by adding the prelimbic area (represented by dashed lines in Figure 7). Finally, although additional counts were made in the medial dorsal thalamic nucleus and the anterior thalamic nuclei, these areas could not be incorporated into acceptable models.[Fig-anchor fig7]

#### Comparison of networks for recency memory and recognition memory

To determine whether the network models calculated for each task (recognition memory and recency memory) are qualitatively different from one another, the data from the recency test group and recognition group were initially stacked on the optimum recognition memory model. The procedure yielded a model of poorer fit when structural weights were constrained to be the same for both groups than when the structural weights of all paths were free to vary between the groups, χ^2^(4) = 14.0, *p* = .015, indicating that there is a significant difference between the groups. Furthermore, data from the recency test group and recognition group were then stacked on the initial (common) part of the optimum recency test group simplex model (perirhinal cortex to lateral entorhinal cortex and then onto CA1); again a significant difference was found between the model in which structural weights were free to vary and the model which constrained the structural weights to be the same for both groups, χ^2^(2) = 9.17, *p* = .01. Together, these dissociations demonstrate that when animals are performing a recency task the Fos data do not fit the model obtained for recognition memory task and vice versa.

### Discussion

The present study used the expression of the immediate-early gene, c-*fos*, to map neuronal activity associated with object recency and object recognition memory in rats. Although c-*fos* activity can only provide an indirect measure of neuronal activity (Herdegen, 1996; Herrera & Robertson, 1996; Kovács, 2008), blocking c-*fos* expression in the perirhinal cortex does disrupt long-term object recognition memory (Seoane et al., 2012); that is, c-*fos* activity has an integral role in the stabilization of recognition information. This finding builds on the consistent finding of raised c-*fos* activity levels in caudal perirhinal cortex when rats see novel stimuli (Albasser et al., 2013; Albasser, Poirier, & Aggleton, 2010; Wan et al., 1999, 2004; Warburton et al., 2005, 2003; Zhu, Brown, McCabe, & Aggleton, 1995, 1996). In those studies in which the animals could actively investigate novel and familiar test objects and, thereby, demonstrate their recognition memory, additional increased hippocampal activity has been found (Albasser et al., 2013; Albasser, Poirier, & Aggleton, 2010). By applying SEM to the counts of Fos-positive cells, models have been derived of interlinked medial temporal activity associated with recognition memory (Albasser, Poirier, & Aggleton, 2010). The present study extended these investigations by deriving activity models for recency memory, that is, temporal order memory. These models could then be compared with those for recognition memory.

The recency test group in Experiment 1b was able to discriminate between familiar objects separated by an interval of 110 min. In contrast, the rats in the recency control group were given pairs of familiar objects that were separated by at most 1 min (but often, only seconds). In this way, it was possible to match very closely the sensorimotor experiences of the two groups. The recency control group showed no evidence of being able to discriminate between the test objects. Thus, although the control rats were given a recency problem, the behavioral evidence indicated that the rats in this condition treated the objects as though they were temporally indistinguishable. An important assumption is that the recency test group relied on recency memory in Experiment 1b. To examine this assumption, all rats were first tested on their ability to distinguish novel from familiar objects (Experiment 1a). This initial, experiment used the same retention delays for the familiar objects as those subsequently used for the recency memory tests in Experiment 1b. The ability of the rats to recognize novel objects in Experiment 1a confirmed that rats could retain familiarity information over the time intervals subsequently used in the recency tests. This finding is important as it shows that the recency tests did involve discriminating between two familiar objects. A caveat is that it cannot be proved whether this assumption applies to each individual trial as the results from Experiment 1a reflect cumulative data from multiple trials, while the objects, by necessity, were different from those in Experiment 1b.

In some key respects, the best fitting c-*fos* networks for the recency test and recency control conditions were similar, presumably reflecting the fact that both involved objects previously made familiar. Both networks involved connections from the perirhinal cortex to lateral entorhinal cortex and CA1, while neither the dentate gyrus nor CA3 could be incorporated into models with acceptable fit. The two networks did, however, differ as the recency test model was linear while the recency control model had multiple pathways emerging from the perirhinal cortex (Figure 6). As a consequence, the recency control model contained fewer degrees of freedom, so limiting its ability to pick out the key pathways. Particularly striking, however, was the way in which both of the networks involving familiar stimuli contrasted with the best-fitting model associated with the discrimination of novel stimuli; that is, recognition memory (Experiment 2). For recognition memory, the optimum network had parallel pathways from the lateral entorhinal cortex; one to the dentate gyrus and another to CA1 (Figure 7). The dentate gyrus pathway next involved CA3, which then converged on CA1. Thus, it appears that the presence of novelty led to different patterns of hippocampal engagement, involving greater parahippocampal interaction with the dentate gyrus and CA3.

Performance in the recency test condition (D1, D2) correlated with the Fos-positive cell counts in several ROIs, most notably within the parahippocampal region and hippocampus. Significant positive correlations with recency performance were found for both areas 35 and 36 (perirhinal cortex), with the correlations with caudal perirhinal cortex typically being the highest. Other positive correlations between recency discrimination performance and Fos counts were found in area Te2 and the lateral entorhinal cortex. Given the modest numbers of rats in the Recency test group and the total numbers of correlations, there is a danger of Type I errors because of multiple comparisons. There was, however, a strong clustering of significant correlations in the regions of previously linked to recency memory. For example, the recency memory link with perirhinal cortex builds onto considerable evidence highlighting the role of this area in processing complex visual information, including information to help resolve both visual recognition and visual recency problems (Barker et al., 2007; Barker & Warburton, 2011a; Brown & Aggleton, 2001; Brown & Xiang, 1998; Fahy, Riches, & Brown, 1993; Hannesson, Howland, et al., 2004; Winters et al., 2008). The present correlations extend this association to visual area Te2 and the entorhinal cortex, regions that are strongly interconnected with the perirhinal cortex (Burwell & Amaral, 1998; Furtak et al., 2007). Area Te2 has previously been implicated in object recognition memory, with evidence from electrophysiology (Zhu, Brown, & Aggleton, 1995), lesion studies (Ho et al., 2011), and c-*fos* expression studies (Albasser, Poirier, & Aggleton, 2010; Wan et al., 1999, 2004; Zhu, Brown, McCabe, & Aggleton, 1995; Zhu, McCabe, Aggleton, & Brown, 1996, 1997). The present, additional links with recency memory builds on previous electrophysiological evidence that Te2 cells can signal temporal order differences (Zhu, Brown, & Aggleton, 1995).

The best-fitting models for Recency Test group involved the pathways from the caudal perirhinal cortex to lateral entorhinal cortex and, thence to CA1. This hippocampal involvement is supported by the finding of significant correlations between the D1 and D2 scores and the Fos-positive cell counts in CA1, CA3 and subiculum. This regional pattern accords with lesion studies, which have not only shown the importance of the hippocampus for object recency memory (Barker et al., 2007; Barker & Warburton, 2011a), including when tested in the bow-tie maze (Albasser et al., 2012), but have also shown that this hippocampal involvement depends on the perirhinal cortex (Barker & Warburton, 2011b; Warburton & Brown, 2010). In this respect, the c-*fos* recency networks also bear a strong similarity to networks derived from a group of rats (“familiarity” control) examined in a previous study of object recognition (Albasser, Poirier, & Aggleton, 2010). In that study, the Familiarity control rats were simultaneously shown a highly familiar object (from the previous trial) and an object that is less recent but still highly familiar (used in all previous sessions). Consequently, the familiarity control involved recency judgments (Albasser, Poirier, & Aggleton, 2010). It is therefore striking that in all three behavioral conditions involving familiar objects (recency test, recency control, familiarity control; Albasser, Poirier, & Aggleton, 2010), the optimum pathway model involved direct lateral entorhinal cortex to CA1 interactions but not lateral entorhinal to dentate gyrus (or CA3) interactions. These same patterns are echoed in a *zif*268 study of spatial learning, as familiar spatial problems preferentially engaged entorhinal cortex to CA1 pathways, while more novel spatial problems engaged pathways from entorhinal cortex to the dentate gyrus and CA3 (Poirier et al., 2008).

Experiment 2 examined object recognition. The present study involved a new set of rats and also incorporated data from a previous study that used the same protocols (Albasser, Poirier, & Aggleton, 2010). The combination of data for additional modeling is permissible as these analyses are based on the individual correlations between areas and not on absolute counts. The resulting models again highlighted the potential significance of caudal perirhinal cortex for visual object recognition (Albasser et al., 2009; Albasser, Poirier, & Aggleton, 2010; Wan et al., 1999), which contrasts with the rostral perirhinal cortex activation found in tests of object recognition in the dark (Albasser et al., 2013). This rostral-caudal difference is likely to reflect the finding that visual inputs preferentially terminate in caudal perirhinal cortex (Furtak et al., 2007). The increase in power from combining data sets also made it feasible to test models with a greater number of nodes. These more extended models sought to incorporate thalamic and prefrontal areas. The recognition model with the best fit involved an additional projection from entorhinal cortex to prelimbic cortex (Figure 7). This pattern can be contrasted with the recency memory model, which incorporated the subiculum connections with prelimbic cortex (recency test). This model parallels the outcomes of disconnection studies, which link hippocampal and prelimbic contributions for recency memory, but not for recognition memory (Barker & Warburton, 2011a, 2011b).

Together, these activation studies indicate that novelty/familiarity detection in the perirhinal cortex has a downstream impact on hippocampal processing. This notion is central to the gatekeeper hypothesis of perirhinal function (Fernández & Tendolkar, 2006), in which the perirhinal cortex regulates hippocampal processing according to stimulus novelty. The present results refine this model by indicating that novel stimuli are more likely to engage additional dentate gyrus/CA3 processing. This engagement would lead to enhanced learning of associated information, such as stimulus location and context. This prediction is supported by selective lesion studies of the dentate gyrus and CA3 field (Gilbert, Kesner, & Lee, 2001; Hunsaker, Rogers, & Kesner, 2007; Hunsaker, Rosenberg, & Kesner, 2008; Lee, Hunsaker, & Kesner, 2005), as well as c-*fos* activation studies showing the engagement of these particular subfields by novel spatial configurations (Jenkins, Amin, Pearce, Brown, & Aggleton, 2004). In contrast, familiar stimuli should preferentially engage CA1 processing; for example, in recency discriminations. Again, both lesion studies (Gilbert et al., 2001; Hoge & Kesner, 2007; Kesner, Hunsaker, & Ziegler, 2010) and c-*fos* expression studies (Amin, Pearce, Brown, & Aggleton, 2006) have highlighted the importance of the CA1 field for temporal processing. Of particular relevance is the finding that CA1 lesions impaired object recency discriminations that were spared by CA3 lesions (Hoge & Kesner, 2007). This dissociation matches the pattern of data derived from the present c-*fos* expression study.

The influential notion that the dentate gyrus has a particular role in pattern separation (Clelland et al., 2009; Gilbert, Kesner, & DeCoteau, 1998; Hunsaker & Kesner, 2013; Leutgeb, Leutgeb, Moser, & Moser, 2007) could readily be integrated within these network models as pattern separation is likely to be of particular priority for novel stimuli; for example, to help determine their unique attributes for associative learning. This same analysis serves to emphasize how parahippocampal and hippocampal regions cooperate when processing stimulus novelty. The assumption is that both regions work together but in sequentially different ways. The perirhinal cortex processes stimulus identity and, thereby, familiarity (Brown & Aggleton, 2001; Cowell, Bussey, & Saksida, 2010). Hippocampal contributions more closely reflect additional learning (e.g., spatial, temporal information) linked to the target object and, thereby, should be required when solving associative recognition problems (Barker & Warburton, 2011b; but see Langston & Wood, 2010). (The term “associative recognition” refers to when all individual stimuli are familiar but their recombination or changed location creates a novel configuration; Barker & Warburton, 2011b; Mayes, Montaldi, & Migo, 2007.)

The network models for recency memory involved the prelimbic cortex, as well as limbic thalamic nuclei. Prelimbic cortex has repeatedly been implicated in recency memory (Cross, Aggleton, Brown, & Warburton, 2013; DeVito & Eichenbaum, 2011; Hannesson, Howland, et al., 2004, Hannesson, Vacca, Howland, & Phillips, 2004), supporting the models in the present study. In addition, there is disconnection evidence that the prelimbic cortex functions in conjunction with the medial dorsal thalamic nucleus to support recency discriminations (Cross et al., 2013). Other lesion studies have implicated the anterior thalamic nuclei in some forms of recency memory (Dumont & Aggleton, 2013; Wolff, Gibb, & Dalrymple-Alford, 2006). In the present study, the anterior thalamic nuclei were incorporated in the recency test model while the medial dorsal nucleus was incorporated in the recency control model. The implication is that the anterior thalamic nuclei and medial dorsal nuclei have subtly different roles concerning familiar objects. This notion is appealing given their very different properties. In particular, the anterior thalamic nuclei have been repeatedly implicated in episodic memory (Aggleton & Brown, 1999, 2006; Aggleton, Dumont, & Warburton, 2011; Carlesimo, Lombardi, & Caltagirone, 2011), and it could readily be argued that those tests of recency memory which involve registering intervening events, as well the passage of time per se, place added demands on episodic memory (see Eacott & Easton, 2010). In doing so, one would predict a particular link between the hippocampus (subiculum) and anterior thalamic nuclei for such recency problems (but see Dumont & Aggleton, 2013; Wolff et al., 2006).

In summary, tasks involving familiar stimuli (recency memory) result in activation networks that differ appreciably from those networks associated with novel stimuli (recognition memory). Although all of the networks involved the hippocampus, lesion evidence often shows that this structure is not required for successful novelty detection (e.g., Brown, Warburton, & Aggleton, 2010; Forwood et al., 2005; Mumby, 2001; Winters, Forwood, Cowell, Saksida, & Bussey, 2004), although it is consistently critical for recency memory (Agster et al., 2002; Barker et al., 2007; Barker & Warburton, 2011a,2011b; Fortin et al., 2002). Furthermore, recency memory appears especially linked with the CA1 field, with supporting evidence from both lesion studies and the present c-*fos* analyses. The implication is that object novelty is initially detected upstream from the hippocampus and this information then moderates modes of hippocampal processing. This change in the hippocampal processing of novel stimuli can then result in better learning of stimulus attributes via activity in the dentate gyrus and CA3. This additional attribute information can then aid recognition judgments as the associated information may increase the confidence of novel versus familiar discriminations (Eichenbaum, Fortin, Sauvage, Robitsek, & Farovik, 2010). At the same time, the novelty signal itself is often sufficient to guide object recognition and so does not require the integrity of the hippocampus.

## Supplementary Material

10.1037/a0037055.supp

## Figures and Tables

**Table 1 tbl1:** Schematic Diagram Showing the Sequence of Object Presentations in Experiments 1a, 1b, and 2

Trials	1	2	3	4	5	6	7	8	9	10	11	12	13	14	15	16	17	18	19	20
Experiment 1																				
First sample	**A**	**B**	**C**	**D**	**E**	**F**	**G**	**H**	**I**	**J**	**K**	**L**	**M**	**N**	**O**	**P**	**Q**	**R**	**S**	**T**
Phase	**A**	**B**	**C**	**D**	**E**	**F**	**G**	**H**	**I**	**J**	**K**	**L**	**M**	**N**	**O**	**P**	**Q**	**R**	**S**	**T**
Second sample	**a**	**b**	**c**	**d**	**e**	**f**	**g**	**h**	**i**	**j**	**k**	**l**	**m**	**n**	**o**	**p**	**q**	**r**	**s**	**t**
Phase	**a**	**b**	**c**	**d**	**e**	**f**	**g**	**h**	**i**	**j**	**k**	**l**	**m**	**n**	**o**	**p**	**q**	**r**	**s**	**t**
Recognition control (1a)	A	B	C	D	E	F	G	H	I	J	K	L	M	N	O	P	Q	R	S	T
	**α**	**β**	**γ**	**δ**	**ϵ**	**ζ**	**η**	**θ**	**ι**	**ξ**	**κ**	**λ**	**μ**	**υ**	**ο**	**π**	**ς**	**ρ**	**σ**	**τ**
Recency test (1b)	A	B	C	D	E	F	G	H	I	J	K	L	M	N	O	P	Q	R	S	T
	a	b	c	d	e	f	g	h	i	j	k	l	m	n	o	p	q	r	s	t
Recency control (1b)	A	a	C	c	E	e	G	g	I	i	K	k	M	m	O	o	Q	q	S	s
	B	b	D	d	F	f	H	h	J	j	L	l	N	n	P	p	R	r	T	t
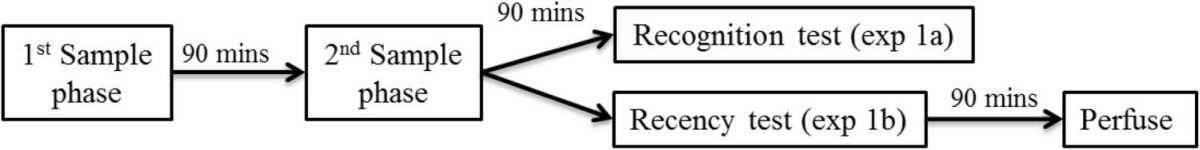																				
Experiment 2																				
Recognition	**A**	**B**	**C**	**D**	**E**	**F**	**G**	**H**	**I**	**J**	**K**	**L**	**M**	**N**	**O**	**P**	**Q**	**R**	**S**	**T**
Memory	—	A	B	C	D	E	F	G	H	I	J	K	L	M	N	O	P	Q	R	R
*Note.* Different objects are represented by different letters and by changes in case (upper or lower). To represent the first presentation of an object (i.e., when novel), the letter is in bold. The table shows the order of object presentation across the different phases of the recognition (Experiment 1a) and recency (Experiment 1b) procedures. The structure of the first two phases for Experiments 1a and 1b were identical. In contrast, Experiment 2 did not involve a separate sample phase prior to the test phase because they were integrated in a single phase. For the two recognition memory tests (Experiments 1a and 2), each test trial comprised one familiar and one novel object. For the recency conditions (Experiment 1b), each test trial comprised two familiar objects from different times in the past.																				

**Table 2 tbl2:** Experiment 1b: Interarea Correlations of c-Fos Counts and Behavioral Measures (Exploration D1, and D2)

Recency test group																				
		Explo	D1	D2	CA1	CA3	DG	lEnto	mEnto	pRhinal	Subi	MD	Prelimbic	TE2	ATN	Reuniens	rPrh	mPrh	cPrh	Audp
Explo	*r*		.612	−.213	−.070	.154	−.081	.179	.210	.124	.086	.465	.271	.103	.112	.280	.313	.387	.122	−.305
	*p*		.080	.582	.857	.692	.837	.645	.588	.751	.826	.208	.480	.792	.774	.466	.413	.303	.754	.425
D1	*r*	−.676*		.617	.574	.668*	.394	.718*	.774*	.574	.803**	.334	.484	.657	.378	.717*	.720*	.775*	.780*	.485
	*p*	**.045**		.077	.106	**.049**	.294	**.029**	**.014**	.106	**.009**	.379	.187	.054	.316	**.030**	**.029**	**.014**	**.013**	.186
D2	*r*	−.655	.933**		.790*	.677*	.597	.758*	.715*	.677*	.858**	.019	.290	.773*	.310	.650	.545	.588	.858**	.879**
	*p*	.056	**<.001**		**.011**	**.045**	.090	**.018**	**.030**	**.045**	**.003**	.961	.449	**.014**	.417	.058	.129	.096	**.003**	**.002**
CA1	*r*	.583	.004	−.080		.851**	.932**	.897**	.682*	.678*	.830**	.254	.193	.662	.398	.648	.391	.392	.626	.558
	*p*	.100	.991	.837		**.004**	**<.001**	**.001**	**.043**	**.045**	**.006**	.509	.620	.052	.289	.059	.298	.297	.071	.118
CA3	*r*	.535	−.249	−.267	.659		.863**	.819**	.594	.736*	.829**	.435	.528	.845**	.760*	.907**	.610	.665	.556	.575
	*p*	.138	.519	.488	.053		**.003**	**.007**	.092	**.024**	**.006**	.242	.144	**.004**	**.017**	**.001**	.081	.051	.120	.105
DG	*r*	.616	−.085	−.188	.906**	.769*		.771*	.440	.679*	.671*	.257	.103	.592	.512	.623	.202	.251	.388	.344
	*p*	.078	.827	.629	**.001**	**.015**		**.015**	.236	**.044**	**.048**	.505	.792	.093	.159	.073	.603	.514	.302	.365
lEnto	*r*	.654	−.097	−.131	.917**	**.665**	.778*		.705*	.586	.772*	.495	.379	.787*	.360	.776*	.645	.651	.737*	.589
	*p*	.056	.804	.737	**.001**	**.050**	**.014**		**.034**	.097	**.015**	.176	.314	**.012**	.341	**.014**	.061	.057	**.023**	.095
mEnto	*r*	.317	.191	.308	.664	.436	.548	.662		.582	.840**	.393	.520	.529	.175	.512	.634	.528	.656	.656
	*p*	.406	.622	.419	.051	.240	.126	.052		.100	**.005**	.295	.151	.144	.652	.158	.067	.144	.055	.055
pRhinal	*r*	.688*	−.191	−.130	.859**	.552	.772*	.875**	.835**		.741*	.181	.318	.701*	.335	.671*	.287	.419	.568	.456
	*p*	**.040**	.623	.738	**.003**	.123	**.015**	**.002**	**.005**		**.022**	.641	.404	**.035**	.378	**.048**	.454	.262	.111	.218
Subi	*r*	.655	−.202	−.170	.649	.731*	.561	.748*	.686*	.679*		.148	.490	.757*	.455	.741*	.647	.643	.815**	.755*
	*p*	.055	.602	.661	.059	**.025**	.116	**.020**	**.041**	**.044**		.704	.181	**.018**	.219	**.022**	.060	.062	**.007**	**.019**
MD	*r*	.909**	−.755*	−.702*	.484	.631	.512	.592	.389	.651	.771*		.638	.394	.324	.500	.549	.466	−.012	.116
	*p*	**.001**	**.019**	**.035**	.186	.069	.158	.093	.300	.058	**.015**		.065	.295	.395	.171	.126	.207	.975	.766
Prelimbic	*r*	.691*	−.273	−.194	.734*	.706*	.647	.727*	.770*	.796*	.917**	.782*		.649	.507	.693*	.865**	.772*	.326	.583
	*p*	**.039**	.478	.618	**.024**	**.033**	.060	**.027**	**.015**	**.010**	**<.001**	**.013**		.058	.163	**.038**	**.003**	**.015**	.392	.099
TE2	*r*	.345	−.132	−.021	.510	.712*	.372	.633	.701*	.586	.858**	.591	.820**		.601	.964**	.779*	.873**	.751*	.766*
	*p*	.363	.734	.958	.161	**.031**	.324	.067	**.035**	.097	**.003**	.094	**.007**		.087	**<.001**	**.013**	**.002**	**.020**	**.016**
ATN	*r*	.906**	−.719*	−.594	.472	.638	.483	.585	.420	.656	.767*	.961**	.813**	.619		.732*	.500	.578	.142	.393
	*p*	**.001**	**.029**	.092	.200	.064	.187	.098	.260	.055	**.016**	**<.001**	**.008**	.076		**.025**	.171	.103	.716	.295
Reuniens	*r*	.676*	−.267	−.325	.801**	.915**	.790*	.826**	.550	.694*	.893**	.755*	.850**	.775*	.721*		.795*	.889**	**.651**	**.641**
	*p*	**.045**	.487	.393	**.009**	**.001**	**.011**	**.006**	.125	**.038**	**.001**	**.019**	**.004**	**.014**	**.028**		**.010**	**.001**	.058	.063
rPrh	*r*	.251	−.464	−.265	−.259	.306	−.143	.026	.162	.106	.352	.496	.210	.461	.503	.196		.943**	.651	.734*
	*p*	.515	.208	.491	.501	.423	.713	.946	.676	.787	.352	.175	.587	.212	.168	.614		**<.001**	.058	**.024**
mPrh	*r*	.528	−.150	−.024	.559	.746*	.550	.742*	.756*	.742*	.817**	.649	.737*	.830**	.679*	.752*	.612		.714*	.692*
	*p*	.144	.701	.950	.118	**.021**	.125	**.022**	**.018**	**.022**	**.007**	.059	**.024**	**.006**	**.044**	**.020**	.080		**.031**	**.039**
cPrh	*r*	.661	−.497	−.475	.529	.605	.601	.546	.627	.733*	.698*	.836**	.750*	.608	.729*	.701*	.454	.684*		.733*
	*p*	.052	.174	.197	.143	.084	.087	.128	.071	**.025**	**.037**	**.005**	**.020**	.083	**.026**	**.035**	.219	**.042**		**.025**
Audp	*r*	.000	−.043	.061	.059	.338	−.139	.150	.303	.046	.600	.288	.537	.781*	.336	.419	.306	.362	.194	
	*p*	.999	.912	.877	.880	.373	.721	.700	.428	.907	.088	.452	.136	**.013**	.376	.262	.423	.338	.616	
Recency control group																				
*Note.* Significant correlations (uncorrected) are in bold. See Figure 2 for abbreviations and Supplemental Table 1 for all correlations.																				
* *p* < .05. ** *p* < .01.																				

**Table 3 tbl3:** Experiment 2: Interarea Correlations of c-Fos Counts and Behavioral Measures (Exploration, D1 and D2)

Recognition group														
		Explo	D1	D2	CA1	CA3	DG	lEnto	MD	Prelimbic	TE2	ATN	cPrh	Audp
Explo	*r*		.741*	.304	−.094	−.142	−.458	−.127	−.048	−.224	.320	.029	.050	.187
	*p*		**.035**	.465	.825	.737	.254	.765	.910	.594	.439	.945	.906	.658
D1	*r*	.738**		.857**	−.005	.061	−.225	.166	.228	−.012	.514	.243	.451	.536
	*p*	**<.001**		**.007**	.990	.887	.593	.695	.588	.977	.193	.563	.262	.171
D2	*r*	.116	.747**		−.009	.128	−.037	.249	.420	.068	.451	.359	.554	.566
	*p*	.647	**<.001**		.983	.763	.932	.552	.300	.872	.262	.383	.154	.144
CA1	*r*	.351	.245	−.051		.954**	.845**	.818*	.240	.599	.575	.347	.308	.349
	*p*	.153	.326	.841		**<.001**	**.008**	**.013**	.567	.117	.136	.400	.458	.397
CA3	*r*	−.362	−.459	−.356	.221		.879**	.768*	.383	.519	.576	.432	.296	.289
	*p*	.140	.055	.147	.377		**.004**	**.026**	.349	.187	.135	.285	.477	.488
DG	*r*	−.424	−.394	−.184	.166	.883**		.620	.294	.460	.243	.308	.128	.083
	*p*	.079	.106	.465	.510	**<.001**		.101	.480	.251	.562	.458	.763	.846
lEnto	*r*	.516*	.568*	.260	.778**	−.**274**	−.225		−.030	.905**	.805*	.055	.782*	.789*
	*p*	**.028**	**.014**	.297	**<.001**	**.271**	.369		.943	**.002**	**.016**	.897	**.022**	**.020**
MD	*r*	−.023	−.247	−.366	.185	**.461**	.249	−.193		−.407	−.021	.959**	−.244	−.206
	*p*	.928	.324	.135	.463	**.054**	.318	.444		.317	.960	**<.001**	.560	.625
Prelimbic	*r*	.479*	.442	.119	.616**	−.**227**	−.275	.857**	−.130		.723*	−.359	.829*	.780*
	*p*	**.044**	.066	.639	**.006**	**.364**	.269	**<.001**	.606		**.043**	.383	**.011**	**.022**
TE2	*r*	.331	.213	−.047	.548*	.544*	.536*	.459	.153	.404		−.006	.823*	.811*
	*p*	.179	.397	.854	**.019**	**.020**	**.022**	.055	.544	.097		.989	**.012**	**.015**
ATN	*r*	−.073	−.179	−.194	.187	.752**	.648**	−.230	.584*	−.198	.451		−.242	−.139
	*p*	.773	.478	.441	.458	**<.001**	**.004**	.359	**.011**	.430	.060		.563	.743
cPrh	*r*	.388	.481*	.242	.511*	**.009**	−.029	.750**	.011	.736**	.628**	−.097		.965**
	*p*	.111	**.044**	.334	**.030**	**.971**	.908	**<.001**	.964	**.001**	**.005**	.701		**<.001**
Audp	*r*	−.003	−.200	−.329	.213	.766**	.729**	−.018	.417	.042	.770**	.643**	.392	
	*p*	.990	.427	.183	.396	**<.001**	**.001**	.942	.085	.869	**<.001**	**.004**	.108	
Combined data with Albasser et al., 2010														
*Note.* The top right correlations are from the present data. The bottom left correlations are from when the present data are combined with that from Albasser et al. 2010. Significant correlations (uncorrected) are in bold. See Figure 2 for all abbreviations and Supplemental Table 2 for all correlations.														
* *p* < .05. ** *p* < .001.
