# Supplementary material for: Contrasting Networks for Recognition Memory and Recency Memory Revealed by Immediate-Early Gene Imaging in the Rat
Source: Behav Neurosci. 2014 Jun 16;128(4):504–22. doi: 10.1037/a0037055 (PMC4105319; doi:10.1037/a0037055)
Supplement: Supplementary file 1 [file BNE-BNE3-Kinnavane20131498-R-Final.docx]

Supplementary Table1: Recency memory and c-*fos* (Exp. 1b). Inter-area correlations of c-*fos* counts and behavioral measures (exploration, D1 and D2) for all areas.

Supplementary Table 2: Recognition memory and c-*fos* (Exp. 2). Inter-area correlations of c-*fos* counts and behavioral measures (exploration, D1 and D2) for all areas.
